# Supplementary material for: Timing mechanism of sexually dimorphic nervous system differentiation
Source: eLife. 2019 Jan 1;8:e42078. doi: 10.7554/eLife.42078 (PMC6312707; doi:10.7554/eLife.42078)
Supplement: Supplementary file 1. [file elife-42078-supp1.docx]

**Supplementary Dataset 1: List of sexually dimorphic, but *lin-29A* independent locomotory features.**

| **Sexually dimorphic features**  **not affected by *lin-29A* absence** | **Type** |
| --- | --- |
|  |  |
| *Absolute forward tail bend (degrees)* | *posture* |
| *Track length(microns)* | *path* |
| *Eccentricity (no units)* | *path* |
| *Bend count (counts)* | *motion* |
| *Coils Frequency (Hz)* | *motion* |
| *Coil Time (seconds)* | *posture* |
| *Absolute tail to head orientation (degrees)* | *posture* |
| *Absolute eigen projection 1* | *posture* |
| *Absolute eigen projection 2* | *posture* |
| *Absolute eigen projection 4* | *posture* |
| *Forward head-tip speed (microns/seconds)* | *motion* |
| *Forward head speed (microns/seconds)* | *motion* |
| *Forward tail speed (microns/seconds)* | *motion* |
| *Absolute head tip motion direction (degrees/seconds)* | *motion* |
| *Absolute head motion direction (degrees/seconds)* | *motion* |
| *Absolute tail motion direction (degrees/seconds)* | *motion* |
| *Absolute tail-tip motion direction (degrees/seconds)* | *motion* |
| *Absolute foraging speed (degrees/seconds)* | *motion* |
| *Head crawling frequency (Hz)* | *motion* |
| *Midbody crawling frequency (Hz)* | *motion* |
| *Tail crawling frequency (Hz)* | *motion* |
| *Omega turns frequency (Hz)* | *motion* |
| *Worm dwelling (seconds)* | *path* |
